# Safety and immunogenicity of the quadrivalent HPV vaccine in female Systemic Lupus Erythematosus patients aged 12 to 26 years

**DOI:** 10.1186/1546-0096-11-29

**Published:** 2013-08-07

**Authors:** Arzu Soybilgic, Karen B Onel, Tammy Utset, Kenneth Alexander, Linda Wagner-Weiner

**Affiliations:** 1 University of Illinois, Chicago, USA; 2 University of Chicago, Chicago, IL, USA

**Keywords:** Human papillomavirus, Lupus, Safety, Immunogenicity, Pediatric, Vaccine

## Abstract

**Background:**

Women with SLE have higher rates of persistent human papilloma virus (HPV) infections and precancerous lesions than healthy women. HPV vaccine is safe and effective in healthy females aged 9–26 years. There are limited data on the safety and immunogenicity of HPV vaccine in females with SLE, and none in adolescents with SLE. Our study evaluates the safety and immunogenicity of recombinant quadrivalent HPV vaccine, Gardasil, in adolescents and young women with SLE.

**Methods:**

This is a prospective, open-label study. Exclusion criteria included disease exacerbation within past 30 days; rituximab or cyclophosphamide within 6 months; pregnancy. Vaccine was administered at months 0, 2, and 6. Physical examination, SLEDAI scores and laboratory studies were performed at months 0, 2, 4, 6 and 7. Each patient’s SLEDAI scores and laboratory profile in the year prior to vaccine administration were used as controls for that patient. Primary outcome measures were change in SLEDAI and mean HPV antibody titers.

**Results:**

27 patients, 12 to 26 years, were enrolled; 20 completed the study. Nine had mild/moderate lupus flares. Mean SLEDAI scores decreased from 6.14 pre-vaccination to 4.49 post-vaccination (p = 0.01). Of 12 patients with lupus nephritis, two experienced worsening renal function during/after the study and progressed to renal failure within 18 months of the study. Both had Class IV lupus nephritis with high chronicity scores (≥ 8) on renal biopsies performed within one year prior to study entry. Seropositivity post-vaccine was >94% for HPV 6, 11, 16 and 18.

**Conclusions:**

Quadrivalent HPV vaccine seems generally safe and well tolerated in this series of adolescents and young women with SLE, with no increase in mean SLEDAI scores. Progression to renal failure in two patients was most likely secondary to pre-existing severe renal chronicity and not secondary to HPV vaccination. Immunogenicity to the quadrivalent HPV vaccine was excellent, with the seropositivity rate >94% in all four HPV types.

## Background

Human papilloma virus (HPV) is the most common sexually-transmitted infection in the United States, and it is directly related to the development of cervical cancer [1]. It is estimated that 20 million Americans between 15 to 49 years of age, approximately 15% of the population, have been or are currently infected with HPV. About half of those who are infected with HPV are sexually active adolescents and young adults 15 to 24 years of age [2]. Between 5% and 30% of individuals infected with HPV are infected with more than one HPV type [3,4].

SLE patients were found to have an increased risk of persistent HPV infection compared to healthy females. They also have a higher risk for developing abnormal cervical smears and squamous intraepithelial lesions (SIL) of the cervix [5-7]. A recent study showed that British women with a recent SLE diagnosis had significantly higher levels of HPV infections, abnormal cervical cytology, and SIL. HPV-16 and HPV-18 DNA positivity were not associated with previous or current drug therapy for the SLE patients in this study, suggesting disease-specific factors might cause the high HPV infection and precancerous lesion rates in SLE patients rather than medication-related immunosuppression [8]. However, other studies have shown that women with SLE also have a much higher risk of developing cervical dysplasia if they are on immunosuppressive chemotherapy, such as cyclophosphamide [9-11]. Therefore, it is currently unclear whether these associations are host-related, SLE syndrome specific, or, alternatively, represent a permissive effect of immunosuppressive therapy on increased host susceptibility to high-cancer-risk human HPV infections [12-14].

The quadrivalent human papillomavirus (HPV) vaccine, Gardasil®, is the first vaccine developed to protect against infection with HPV types which are associated with most genital warts and cervical cancer. The vaccine series can be started at 9 years of age. Catch-up vaccination is recommended for 13 through 26 year old females who have not yet received or completed the vaccination series [1].

Because of their increased risk of persistent HPV infections, vaccination against HPV in lupus patients is especially important. There may be a concern that patients with SLE might not develop an adequate immune response to the vaccine because of immune dysfunction related to SLE itself and also the immunosuppression secondary to treatment of SLE. However, many other vaccines, including hepatitis B [15], pneumococcal vaccine [16,17], and influenza vaccine [18], have been shown to be immunogenic in the SLE population. Although these vaccines have found to be generally safe and well tolerated by SLE patients, lupus-like syndrome and other autoimmune phenomena such as induction of lupus anticoagulants, anti-Ro/La and anti-smith antibodies have been reported following various immunizations [19].

There are limited data on the immunogenicity and safety of HPV vaccination in young women with SLE [20] and no studies that evaluate this vaccination in adolescents with juvenile SLE. Our study primarily aims to evaluate the immunogenicity of the quadrivalent HPV vaccine in this patient population and assess its effects on SLE disease activity.

## Methods

### Patient selection

Female SLE patients, 9 to 26 years of age, followed at the pediatric and adult rheumatology clinics at the University of Chicago were invited to participate in the study. All patients fulfilled the revised ACR classification criteria for SLE [21].

Exclusion criteria included: acute exacerbation of disease within past 30 days which resulted in ≥ 3 points increase in SLEDAI, an increase in corticosteroid dose, initiation of a new immunosuppressive medication, or hospitalization; cyclophosphamide or rituximab treatment in the last six months prior to screening; history of allergic disease or reactions likely to be exacerbated by any component of the vaccine; previous administration of any HPV vaccines; pregnancy.

The Institutional Review Board of the University of Chicago approved the study. Informed consent and assent (if the patient was <18 years old) were obtained from all participants. The study was conducted in accordance with the ethical principles as described in the Declaration of Helsinki and consistent with Good Clinical Practice and applicable regulatory requirements, including HIPAA.

### Study protocol

This is an open label, prospective, pre-post intervention study. Potential participants’ records were reviewed for clinical manifestations of SLE, laboratory parameters, SLE Disease Activity Index (SLEDAI) scores, and medications in the one year prior to study entry [22]. Each patient’s SLEDAI scores and laboratory profile in the year prior to vaccine administration were used as controls for that patient. Prior to vaccination, a complete history and physical examination were performed. Patients were scheduled to receive three doses of 0.5 ml of recombinant, quadrivalent HPV vaccine. (Gardasil®, Merck & Co., Inc., USA), with the initial dose given at study entry, and the second and third doses given two and six months after the initial dose, respectively [23]. Patients were observed in the clinic for 30 minutes after each vaccine dose for acute allergic reactions or syncope. HPV vaccine-related side effects were evaluated systematically, including but not limited to pain, erythema, swelling and itching at the injection site, fever, fatigue, arthralgias, myalgias, headache, dizziness, syncope, influenza-type symptoms, and signs and symptoms of an acute allergic reaction including dyspnea and hives. At study entry and at months 2, 4, 6 and 7, patients were clinically evaluated, and physical examination and laboratory data were recorded. Clinical manifestations of SLE were carefully analyzed and a SLEDAI score was calculated at each of the clinic visits.

### Laboratory evaluation

Laboratory tests performed at months 0, 2, 4, 6 and 7 included complete blood count (CBC), complete metabolic panel (CMP), C3 and C4 complement levels, erythrocyte sedimentation rate (ESR), C-reactive protein (CRP), anti-ds-DNA antibodies, urinalysis, and urine protein/creatinine ratio. Anti-cardiolipin antibodies, β2-Glycoprotein antibodies, lupus anti-coagulant, anti-RNP and anti-Smith antibodies, and HPV antibodies were evaluated at months 0 and 7 of the study. All patients underwent urine pregnancy testing prior to administration of each quadrivalent HPV vaccine dose. Patients who were found to be pregnant were instructed to defer completion of their vaccination regimen until resolution of pregnancy and were removed from the study.

Antinuclear antibodies were detected by indirect Immunofluorescence using HEp-2 cells as substrate. Anti-ds-DNA antibody was detected using IFA (neg <1:10 titer) [24]. Anti-cardiolipin (neg <15 GPL, <12.5 MPL, <12.0 APL Units) and anti-β2-glycoprotein (β2GP) antibodies (neg <20U/ml for IgG, IgM, IgA) were assessed by standardized enzyme-linked immunosorbent assay [25], lupus anti-coagulant by DRVVT (neg <40.4 sec) and PTTLA (neg <44.0 sec) and anti- RNP and anti-Smith antibodies (neg <20 units) by single antigen EIAs (Inova Diagnostics).

Type- specific immunoassays with type-specific standards were used to assess immunogenicity. These assays measured antibodies against neutralizing epitopes for each vaccine HPV type (HPV 6, 11, 16 and 18). The immunogenicity analyses were conducted in a per-protocol immunogenicity (PPI) patient population. This population consisted of individuals who were seronegative to the relevant HPV types at enrollment, received all three vaccinations, and did not deviate from the study protocol in ways that could interfere with the effects of the vaccine. Immunogenicity was measured by the percentage of individuals who converted from seronegative to seropositive for antibodies against the relevant vaccine HPV type, and the cLIA (Competitive Luminex Immunoassay) Geometric Mean Titer (GMT, reported as mili-Merck units (mMu)/ml) [26]. GMTs were measured at month-7. HPV antibody titers were determined by test kits, and cutoff values for seropositivity were titers at or above 20, 16, 20 and 24 mMU/mL for HPV 6, 11, 16 and 18 respectively.

### Statistical analysis

Results are presented as mean plus-minus standard deviation (SD) and percentages. Pre-post changes in SLEDAI and other variables (ESR, CRP, C3, C4) were analyzed using paired t-tests. Anti-ds-DNA antibody titers were first log_10_ transformed (initial 0 titers were scored as 0 post transformation). Each patient’s pre-treatment and post-treatment values were averaged, and the pre-post changes analyzed using both a paired t-test and a nonparametric, Wilcoxon signed rank test. Statistical significance was set as P < 0.05 and 95% confidence intervals were determined.

## Results

27 subjects, ages 12 to 26 years, were enrolled in the study. 27, 25 and 20 subjects received one, two and three doses of HPV vaccine, respectively.

### Demographics and clinical characteristics

22 of 27 patients had juvenile SLE, diagnosed before the age of 16 years. The mean age at enrollment was 20.5 years and mean disease duration was 3.5 years. At the time of study entry, seven patients were ≤ 18 years of age. 14 subjects (51.8%) were African-American, 11 (40.7%) were Caucasian (non-Hispanic), and 2 (7.4%) were Hispanic. Patients were taking the following medications to treat their SLE: hydroxychloroquine (100%); prednisone (59.2%); mycophenolate mofetil (33.3%); azathioprine (33.3%); methotrexate (22.2%). The mean prednisone dose was 12.6 mg (range 0–36). In the past, 14.8% and 18.5% had received cyclophosphamide and rituximab, respectively. Organ system involvement at study entry: musculoskeletal 88.8%, hematologic 51.8%, renal 44.4%, cutaneous 40.7%, neuropsychiatric 37%, serositis 25.9%, cutaneous vasculitis 14.%, cardiovascular 7.4%, pulmonary 3.7%.

### SLEDAI scores

In the 20 patients who completed the study, there was a significant reduction in the mean SLEDAI scores from 6.14 pre-vaccination to 4.49 post-vaccination at month 7 (p- 0.010; 95% CI:-2.85 to −0.44) (Table 1). The two patients who withdrew from the study because of increasing arthralgias had no increase in their SLEDAI scores of at the time of study withdrawal or at their follow-up visits.

**Table 1:** Pre-vaccine and post-vaccine SLEDAI, laboratory values and prednisone doses of those who completed the study

**Variable**	**n**	**Pre-Mean (SD)**	**Post- Mean (SD)**	**95% CI**	**p-value**
SLEDAI	20	6.14 (3.7)	4.49 (2.8)	(−2.85, -0.44)	0.010
Anti-ds-DNA*	16	1.67 (0.28)	1.49 (0.25)	(−0.46, 0.09)	0.17
ESR mm/hr	17	40.9 (21.4)	41.2 (25.6)	(−6.7, 7.4)	0.91
CRP mg/L	15	4.48 (3.53)	3.30 (1.72)	(−2.88, 0.52)	0.16
C3 mg/dl	*20*	92.3 (29.9)	93.0 (26.0)	(−7.4, 8.7)	0.86
C4 mg/dl	*20*	18.2 (10.0)	20.1 (12.4)	(−1.8, 5.7)	0.30
Prednisone (mg) dose	*16*	12.6 (10.8)	7.4 (5.9)	(−8.5, -1.9)	0.005

*Log_10_ transformed.

### Laboratory data

Anti-ds-DNA antibody titers did not change significantly post-vaccination (p = 0.17 by t-test; p = 0.25 by Wilcoxon test). Levels of C3, C4, ESR and CRP remained stable. (Table 1) The mean prednisone dose decreased significantly over the course of the study (p = 0.005).

### Induction of autoantibodies

None of patients who were negative for anti-RNP, anti-Smith, anti-cardiolipin or anti-β2-glycoprotein antibodies at study entry developed them during the study. Lupus anticoagulant (LA) was present in 3 patients at entry; in one of these patients, LA became negative by end of study. Two patients who were LA negative at study entry developed LA by month 7.

### Withdrawals

7 patients did not complete the study. Two patients moved out of the state, two chose to withdraw secondary to increased arthralgias after the second vaccine dose, two were discontinued from the study secondary to pregnancy, and one was lost to follow-up.

### Pregnancies

A total of three subjects became pregnant during the study. One became pregnant after the first vaccine, but elected to terminate the pregnancy for personal reasons. She then completed the three-dose vaccine schedule. Two patients became pregnant after the second dose of HPV vaccine, and these patients decided to continue with their pregnancies and did not receive the third dose. Both these pregnancies resulted in healthy newborns without any noted congenital defects.

### Adverse events

None of the patients had acute allergic, local or vasovagal reactions after receiving HPV vaccine. Nine of 27 patients (33.3%) had a mild-moderate flare during the study period, typically with symptoms similar to those they experienced in flares before vaccine administration. Five had arthralgias, 4 had rash, 2 had pleuritis, and 1 had peripheral neuropathy. One of the patients with rash had a severe cutaneous flare after sun exposure, three months after her second HPV vaccine. She was treated with rituximab as this medication had successfully controlled her lupus disease activity a few years earlier. Of 12 patients with history of lupus nephritis, two of four with Class IV nephritis experienced worsening renal function during/after the study and progressed to renal failure within 18 months of the completion of the study. Both patients had renal biopsies performed during the year prior to study entry which showed marked glomerular sclerosis, interstitial loss and tubular necrosis. One had a chronicity index (CI) of 8 out of 12, and the other had a CI of 10 out of 12.

### Immunogenicity

Of the 20 patients who completed the study, 16 had samples available at month 7 for analysis. Seropositivity rates after three doses of the vaccine were 94.4%, 100%, 100%, and 94.4% for HPV 6, 11, 16 and 18, respectively (Table 2). One patient had no antibody response to HPV types 6 and 18 at month 7, and responses to 11 and 16 were positive but very low, with titers of 75 and 65 mMu/ml, respectively. This was the patient who received rituximab between the second and third vaccine doses.

**Table 2:** Immunogenicity to HPV Vaccine

**HPV genotype**	% Seropositivity *	**95% CI §**	**cLIA GMT mMu/ml † (95% CI)**
Anti-HPV 6	**94.4**	(72.7, 99.9)	427.0 (203.4, 903.4)
Anti-HPV 11	**100**	(81.5, 100)	596.7 (303.6, 1173.2)
Anti-HPV 16	**100**	(81.5, 100)	2263.2 (1019.3, 5024.8)
Anti-HPV 18	**94.4**	(72.7, 99.9)	331.9 (159.8, 689.2)

*Seropositivity and anti-HPV values were determined in the 16 patients who completed the vaccine HPV series and had both pre- and post-vaccination serum available for HPV antibody measurement.

§ CI = Confidence Interval.

†cLIA GMT mMu = Competitive Luminex immunoassay, Geometric Mean Titers measured in milli-Merck units.

## Discussion

In this small, non-randomized, non-controlled prospective study, we have shown that the recombinant quadrivalent HPV vaccine, Gardasil, was generally well-tolerated and immunogenic in adolescents and young women with SLE.

In our patients, HPV vaccine did not result in an increase in mean SLEDAI scores. Nine patients had mild/moderate lupus flares during the study, typically with symptoms similar to flares that they experienced during the year before vaccine administration. Flare rates in our study were similar to those in cited by Mok et al. who studied HPV vaccination in young adults with SLE [20]. However, of our 12 patients with history of lupus nephritis, two with Class IV nephritis experienced worsening renal function during and after the study; both progressed to renal failure. It is notable that both patients had high chronicity scores (≥ 8) on renal biopsies performed within one year prior to study entry. Renal failure would be an anticipated event based on the high chronicity scores on the renal biopsy results on both these patients, and therefore, does not appear to be secondary to HPV vaccine administration [27-29]. The 10 other subjects with lupus nephritis had stable renal function during the 7-month study and follow-up period (8–20 months). None of these 10 patients had chronicity scores >4 on biopsy.

Recombinant quadrivalent HPV vaccine was found to be immunogenic in our patient population with seropositivity rates greater than 94% for all four HPV types. This normal immune response to HPV vaccine occurred despite corticosteroid treatment in approximately 60% of our patients. Furthermore, the majority of our subjects were being treated also with either azathioprine or mycophenolate mofetil at time of immunization. The conversion rate to seropositivity in our cohort of young lupus patients was higher than in those SLE patients who received the quadrivalent HPV vaccine in the study by Mok et al. [20]. Seroconversion rate at month 7 in Mok’s study was 74%, 76%, 92% and 75% to subtypes 6, 11, 16 and 18 respectively. Lower seroconversion rates were noted in those patients on both mycophenolate mofetil and prednisolone. They found no significant correlation between HPV antibody response and age at vaccination. However, other studies have shown that post-vaccination GMTs were 2.0 to 2.9 fold higher in younger girls (10–15 years of age) compared with subjects 15–25 years of age [30]. It is conceivable that the combination of older age of subjects in Mok’s study (mean age was 5.3 years older than our population) and immunosuppressive therapy led to a decreased HPV antibody response.

Patients who received cyclophosphamide or rituximab within 6 months of study entry were excluded from our study, as these medications were found to cause an impaired immune response to other vaccinations in SLE patients [31,32]. Our experience, in which the one patient who received rituximab during the vaccine protocol did not develop immunogenicity to HPV, agrees with previous experience of impaired immune response to vaccinations in patients on rituximab and cyclophosphamide.

Given the increased risk of persistent HPV infection and cervical dysplasia in women with SLE, demonstration of immunogenicity to HPV vaccine is especially important. A recent study from Denmark utilizing national databases showed that nearly half of reported malignancies in SLE patients may be HPV-associated [33]. The standardized incidence ratio (SIR) of anal and vaginal/vulvar cancers in SLE patients compared to the general population was 26.9 and 9.1 respectively. In a disease such as SLE, which has increased morbidity from multiple causes, the prospect of being able to provide prophylaxis against one possible cause of morbidity, HPV-associated malignancy, is encouraging. SLE patients and other immunosuppressed populations will need to be followed longitudinally to know whether the HPV vaccine-induced immunogenicity will be efficacious and provide protection over time against HPV infection and its potential malignant sequelae.

There are reports of induction of autoantibodies in patients with SLE after various immunizations [19,34]. In our study, HPV vaccine did not induce production of autoantibodies such as anti-ds-DNA, RNP, Smith, or anti-phospholipid antibodies. It is notable that a recent post-marketing study of quadrivalent HPV vaccine did not show an increased incidence of new autoimmune diseases in healthy women receiving this vaccine [35]. Chao et al. observed almost 190,000 women for 6 months following completion of the vaccine series and found no increase in the incidence of new SLE cases, one of 16 autoimmune conditions monitored.

Limitations of our study were the relatively small sample size in which one-fourth of the subjects withdrew from the study and the fact that this was a non-controlled study. It is possible that the patients who withdrew may have shown an increase in disease activity or a lack of seropositivity to the vaccine series. Although this study had no control group, we used each patient as her own control, comparing disease activity during the study period with disease activity during the year prior to study enrollment.

## Conclusion

HPV vaccine was immunogenic, generally safe and well-tolerated in our patient population of adolescents and young women with SLE. Seropositivity to HPV after three doses of the quadrivalent vaccine was greater than 94% in all four HPV types. This excellent response occurred even though the majority of our patients were on prednisone and other immunosuppressive medication. Given that the increased risk of persistent HPV infection and its complications in women with SLE is well-established, and that quadrivalent HPV vaccine was immunogenic and well tolerated in our small prospective study, administration of the vaccine series to young females (9–26 years) with SLE should be seriously considered. Continued vigilance and clinical studies to assess for a possible negative impact on disease activity in SLE patients receiving HPV vaccine are warranted.

## Competing interests

• Financial support for the work reported on in the manuscript was received in the form of a study grant from Merck Co.& Inc.

• Dr. Kenneth A. Alexander has received consultant fees, and/or honoraria (more than $10,000) from Merck Co&Inc and he is in the Speakers’ bureau and a paid consultant for Merck Vaccines. The authors declare that they have no other competing interests with regard to the work reported.

## Authors’ contributions

AS, LWW, KBO, KAA, TU made substantial contributions to the study conception and design; AS, LWW, KBO participated in acquisition, analysis and interpretation of data; AS, LWW, KBO, KAA, TU made substantial contribution to drafting the article, revising it critically for important intellectual content and made final approval of the version of the article to be published.
